# Deep learning predicts boiling heat transfer

**DOI:** 10.1038/s41598-021-85150-4

**Published:** 2021-03-10

**Authors:** Youngjoon Suh, Ramin Bostanabad, Yoonjin Won

**Affiliations:** 1 Department of Mechanical and Aerospace Engineering, University of California, 5200 Engineering Hall, Irvine, CA 92617-2700 USA; 2 4200 Engineering Gateway, Irvine, USA

**Keywords:** Mechanical engineering, Applied physics, Fluid dynamics

## Abstract

Boiling is arguably Nature’s most effective thermal management mechanism that cools submersed matter through bubble-induced advective transport. Central to the boiling process is the development of bubbles. Connecting boiling physics with bubble dynamics is an important, yet daunting challenge because of the intrinsically complex and high dimensional of bubble dynamics. Here, we introduce a data-driven learning framework that correlates high-quality imaging on dynamic bubbles with associated boiling curves. The framework leverages cutting-edge deep learning models including convolutional neural networks and object detection algorithms to automatically extract both hierarchical and physics-based features. By training on these features, our model learns physical boiling laws that statistically describe the manner in which bubbles nucleate, coalesce, and depart under boiling conditions, enabling in situ boiling curve prediction with a mean error of 6%. Our framework offers an automated, learning-based, alternative to conventional boiling heat transfer metrology.

## Introduction

Boiling is a heat transfer mechanism that utilizes liquid-to-vapor phase transition to dissipate great amounts of heat with minimal temperature difference^1^. Since boiling enables a system to maintain fairly constant surface temperatures during large thermal energy fluctuations, many modern high power density systems such as power plants, power electronics, laser diodes, and photovoltaics rely on boiling for thermal management^2–4^. The energy per unit area (i.e., heat flux) measures how much thermal energy is relieved via boiling and is a critical factor in characterizing boiling heat transfer. For instance, the efficacy of boiling heat transfer can be quantified by either the improvements in the critical heat flux (CHF) or heat transfer coefficient (HTC)^5^, both of which are functions of the boiling heat curves. With the goal of increasing the CHF limit and HTC, extensive prior works have investigated the effects of flow condition^6,7^, surface treatment and design^5,8–13^, and bubble morphology on boiling curves^14^. These past findings suggest that inherent structural characteristics as well as intrinsic material properties significantly affect boiling performance, and therefore the boiling curve.

Quantification of boiling curves has been extensively studied in many theoretical, numerical, or experimental works. Theoretical research on boiling mechanisms provided the foundations for heat flux estimation^15–18^. However, the intrinsic complexity of the dynamic boiling phenomena has limited those theoretical studies to very simplified models^19,20^. With numerical simulations, single to multi-bubble physics are investigated for detailed characterization of heat flux^21–23^. Although direct numerical simulation of the boiling process enables studying dissipated heat flux at local and global scales, the accuracy of these simulations is debatable^22^. Therefore, researchers still heavily rely on experiments to measure the boiling heat flux via, e.g., thermocouples^24^, electrical power input^25,26^, or infrared (IR) techniques^27^. However, these experimental methods are inefficiently connected with visual information, which is a huge downfall for providing a clear description of dynamic boiling physics. In other words, a bridge between measurements and visual information must be built to relate surface design inputs (e.g., surface morphology, material type, and liquid–vapor and liquid–solid interfaces) with boiling curves. Despite the significance of gathering essential visual information, current measurement setups fail to synchronically analyze image data without extensive user involvement, which is not only time-consuming, but also introduces user bias. The drawbacks of conventional measurement techniques motivate devising a non-destructive and automated optical method that can provide in situ heat flux quantification during boiling.

**Figure 1:**
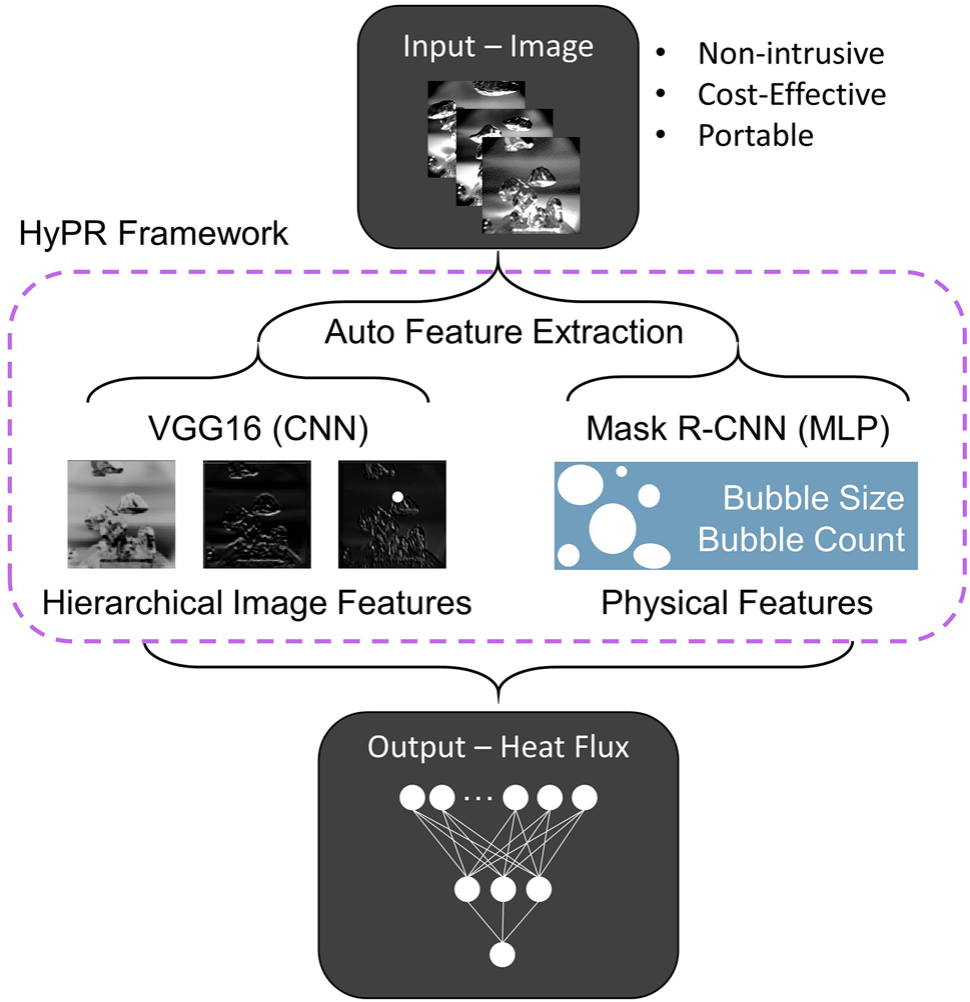
Physics-reinforced learning framework schematic. Remotely acquired images are provided to the framework where cutting-edge convolutional neural network (CNN) architectures and advanced object detection algorithms automatically extract features. The proposed framework learns from a hierarchy of image features as well as physical boiling patterns with the aim of predicting real-time boiling heat flux.

Current advances in deep learning and, in particular, convolutional neural networks (CNNs) have enabled automatic and scalable image analysis for, e.g., object detection^28–31^, classification^32–37^, and even image-based predictions^38–44^. Many CNN-based deep learning frameworks are effective because CNNs emulate the human brain’s natural visual perception mechanism by systematically learning features through multiple operational layers^45^. Image-based deep learning models can play a vital role in fully understanding boiling physics because boiling images are richly embedded with bubble statistics, which are quantitative measurements of the dynamic boiling phenomena^46–48^. Despite the potential for understanding image-based boiling physics via deep learning frameworks, very few attempts have been made to build them. Recent works have developed a framework to classify boiling regimes and to quantify boiling heat transfer^49,50^. However, the boiling experiments in these studies are conducted on one-dimensional (1D) wires, which cannot represent the complex and volatile bubble motions associated with realistic two-dimensional (2D) or three-dimensional (3D) surfaces. Unfortunately, the results from many past models were hard to physically comprehend as they relied on abstract input features such as groups of pixels or principal components^51^. In addition to this, there have been no such an effort to practice machine learning based computer vision link bubble dynamics and boiling processes. For the sake of this effort, we suggest a data-driven framework that predicts boiling heat flux based on high-quality bubble images in real-time (Fig. 1). Our framework conceptualizes state-of-the-art CNNs and object detection algorithms to automatically extract hierarchical image features as well as physics-based bubble statistics to learn inherent boiling physics. By training on these features, the framework not only describes the manner in which the bubbles nucleate and depart under boiling conditions, but also predicts the boiling curves with a mean error of 6% using a small dataset. The framework thereby provides quantitative descriptions of underlying boiling activities that can potentially help discover unknown boiling laws.

## Results

### Hierarchical feature extraction

**Figure 2:**
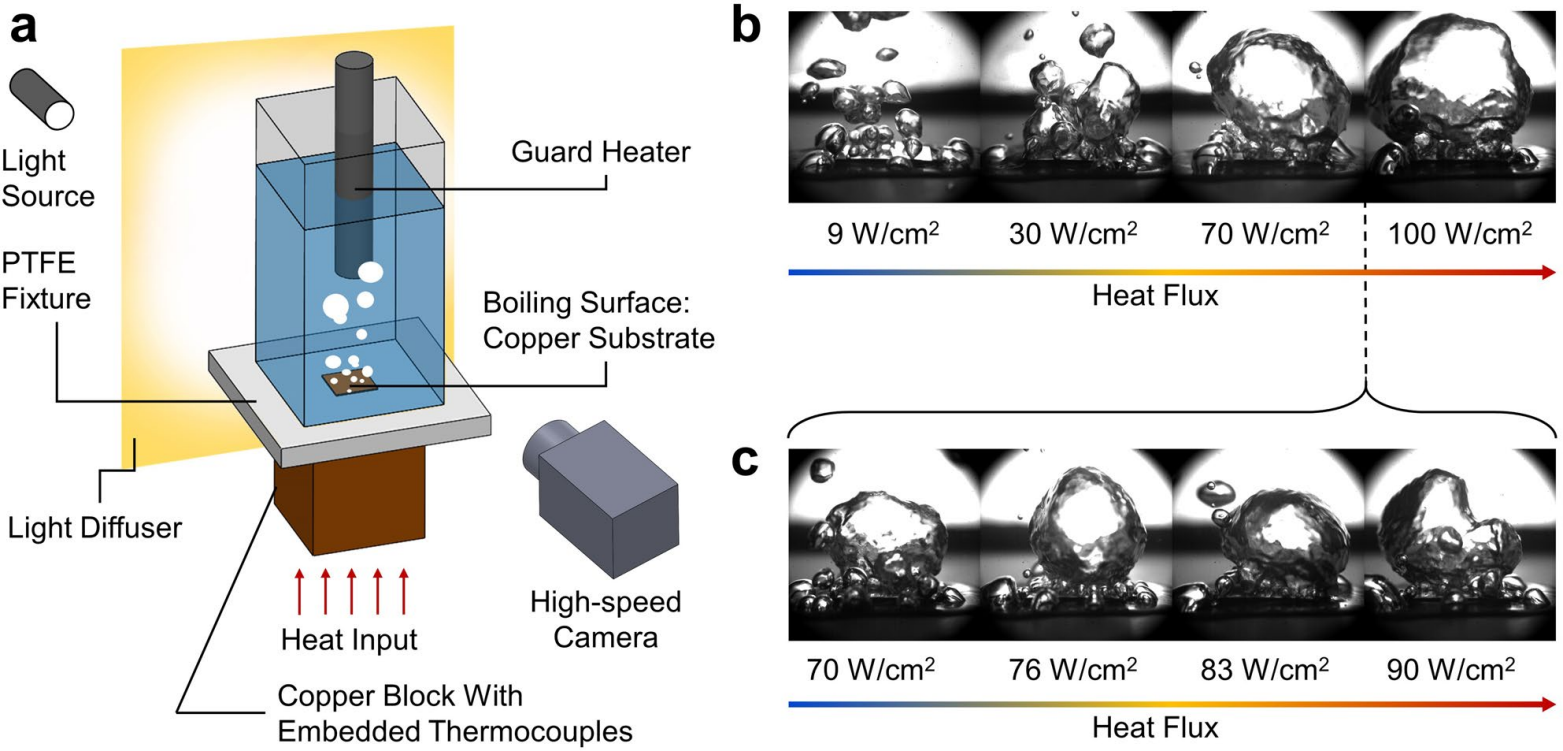
Experimental setup and imaging techniques. (**a**) All pool boiling experiments are conducted on a pool boiling rig with fixed thermal attachment, consistent imaging distance, and an identical plain copper substrate. (**b**) Temporal bubble images captured from the high-speed camera confirm that it is challenging to differentiate bubble changes with small heat flux steps (**c**) However, the changes in bubble appearance become more noticeable when heat flux steps are relatively large.

Our framework primarily employs convolutional neural networks (CNNs) to extract hierarchical image features (see Fig. 2a and Methods for data acquisition). Primitive features such as edges and corners are at the lower levels of this hierarchy, whereas more abstract features (e.g., the existence of a bubble) are at the higher levels. Using these hierarchical features, CNN models can recognize small and critical details in images that the human eye may not perceive. For example, the CNN can differentiate bubble images between relatively small boiling heat flux steps (< 20 W/cm^2^) (Fig. 2b), which is challenging even for the trained eye.. In contrast to the images within similar heat flux ranges, the bubble images display quite distinguishable changes across relatively large heat flux steps (> 20 W/cm^2^) (Fig. 2c), which has been the ranges investigated and analyzed in conventional boiling studies^52^. Deep CNNs will be employed here, because they are known to learn more efficiently than shallow CNNs by naturally integrating incredibly enrichened image features^53^. A robust and easily-trainable deep CNN architecture, VGG16, is selected for this study^54^. Regardless of the high performance of VGG16, the complex and spontaneous nature of the boiling bubble dynamics could still require thousands of images per class to learn from the scratch, leading to a substantial cost of data analysis. A transfer learning technique, called fine-tuning, is performed to retrain a pre-trained CNN network on a specific task, which is bubble image recognition in this case (see Supplementary Information, Fig. S1).

### Physical feature extraction

**Figure 3:**
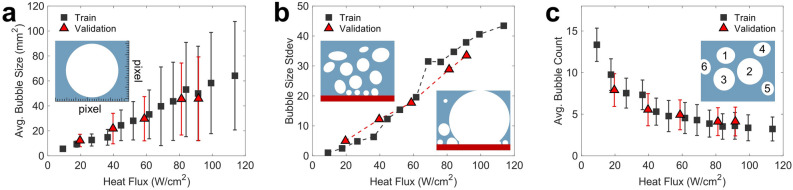
Bubble statistics with varying heat flux. (**a**) The average bubble size and boiling heat flux display a linear relationship. The error bars represent the standard deviation. The inset shows that the bubbles sizes are originally extracted as pixel values. The pixel values are converted to mm^2^ bubble sizes using a commercial program ImageJ and then averaged over the corresponding heat flux. (**b**) The bubble size standard deviation, in turn, characterizes bubble size differences of a given step and also exhibits a near-linear trend. Low heat fluxes have small standard deviations, which means bubbles sizes are relatively uniform. As heat flux increases, the difference becomes small and large bubbles become increasingly noticeable and is reflected in the plot. The bubble size differences for low and high heat fluxes are illustrated in the inset. (**c**) The average bubble count decreases exponentially due to vigorous bubble coalescence as boiling heat flux increases. The inset portrays individual bubbles that are identified and counted.

The second component of our framework employs advanced object detection algorithms to extract pre-determined features (i.e., bubble statistics) that provide clear physical meaning from a group of images. The relationship between bubble statistics (e.g., bubble size and count) and heat flux is well-described in previous studies; higher heat flux increases the wall superheat, thereby facilitating bubble growth and coalescence^55^. However, manual extraction of such detailed information from thousands of images is laborious and time-consuming. To automate image analysis, we employ an instance segmentation model, called Mask R-CNN, to automatically detect and record bubble statistics by measuring individual bubbles in each time frame^56,57^. See Methods Section for Mask R-CNN training process. Figure 3 displays bubble parameters obtained from the Mask R-CNN data analysis as a function of boiling heat flux. As the power is incrementally increased, the number of bubble–bubble interactions increase along with the superheat, displaying a linear correlation between the bubble size and boiling heat flux in Fig. 3a^55^. The error bars in Fig. 3a represent the bubble size deviation, which also linearly correlates with the boiling heat flux in Fig. 3b. The linear increase in bubble size deviation describes the presence of both small and large bubbles in the same image frame at high heat fluxes. It is evident that vigorous bubble coalescing events expedites bubble growth and continuous bubble genesis on heated surfaces. In contrast to the linear increase in bubble size, the average bubble count per frame within one heat flux step exponentially decreases as heat flux increases in Fig. 3c, due to the active bubble coalescence events. The measurements are performed on the train and validation datasets, as described in the Methods Section. The good agreement between those datasets in Fig. 3a–c implies that the bubble statistics are repeatable and thus suitable to predict the boiling curves.

The bubble statistics are then processed through multi-layer perceptron (MLP) neural networks, where feature weights are adjusted to learn boiling physics. The MLP network is implemented because, unlike CNNs, the Mask R-CNN model can only extract features and therefore needs an additional network to train them. The MLP neural networks use a group of 250 images (collected over a few seconds) per each heat flux step as the input, whereas individual images per each heat flux step are processed through CNNs. In the next section, we suggest that the averaged bubble statistics can be incorporated in the CNN’s prediction in a hybrid format, to improve the prediction accuracy. Since prediction models are predominantly built around the MLP network, the compiled Mask R-CNN and MLP neural network model are denoted as the MLP model throughout the paper.

### Hybrid physics-reinforced framework

**Figure 4:**
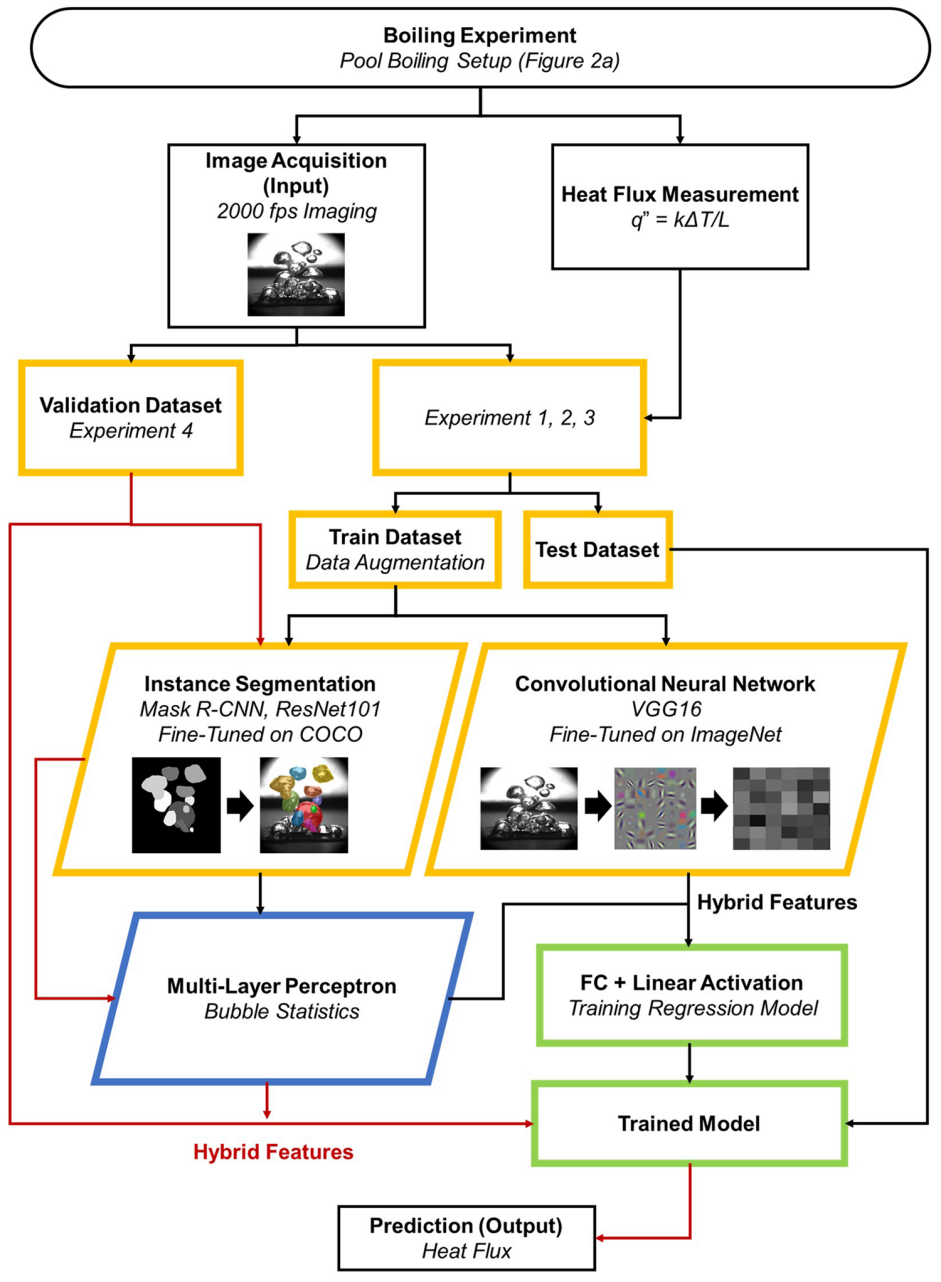
Flow chart for Hybrid physics-reinforced (HyPR) model. The heat flux information is used as labels for the train dataset to provide answers during the training process. All algorithms have no access to heat flux information during testing and validation. The pre-trained Mask R-CNN model extracts bubble statistics and processes the features in the MLP network before being combined with the hierarchical image features extracted by the CNN. A representative plot of an input image, convolutional filters, and fully connected layer for the CNN model is provided, respectively. The validation dataset is used to evaluate the HyPR model’s real-time capability for the boiling heat flux prediction. Yellow, blue, and green frames represent image data, numeric bubble statistics, and the combination of the two data types.

We demonstrate a predictive model for boiling heat flux, denotated as the hybrid physics-reinforced (HyPR) framework, by extending and coupling the two deep learning models that include CNN and MLP models explained in the previous sections. As described in Fig. S2 in detail, the coupling process of CNN and MLP reinforces the model by complementing unique learning strategies of each method; the CNN is capable of recognizing subtle visual alterations (e.g., light diffractions and bubble patterns) without learning the reason why they deviate from original values; whereas the MLP neural network recognizes how bubbles should behave in relation to boiling laws without learning any significant visual variations. Figure 4 showcases how datasets are processed in the HyPR model. Images from the high-speed measurements are distributed into train, test, and validation datasets where only the train datasets are applied with data augmentation for dataset diversification. The augmented images are then simultaneously fed through VGG16 CNN and Mask R-CNN networks where image features and bubble statistics are extracted, respectively. It should be noted that the Mask R-CNN model is already pre-trained to automatically detect and segment bubble images in the previous section. The bubble statistics exported from Mask R-CNN additionally process through MLP neural networks before being concatenated with the CNN outputs. The outputs through the coupled CNN and MLP networks are then fully connected and applied with a linear activation function, which enables the HyPR model to predict continuous heat flux values. The HyPR model is fine-tuned on ImageNet and saved to be assessed with the validation dataset.

### Training results

**Figure 5:**
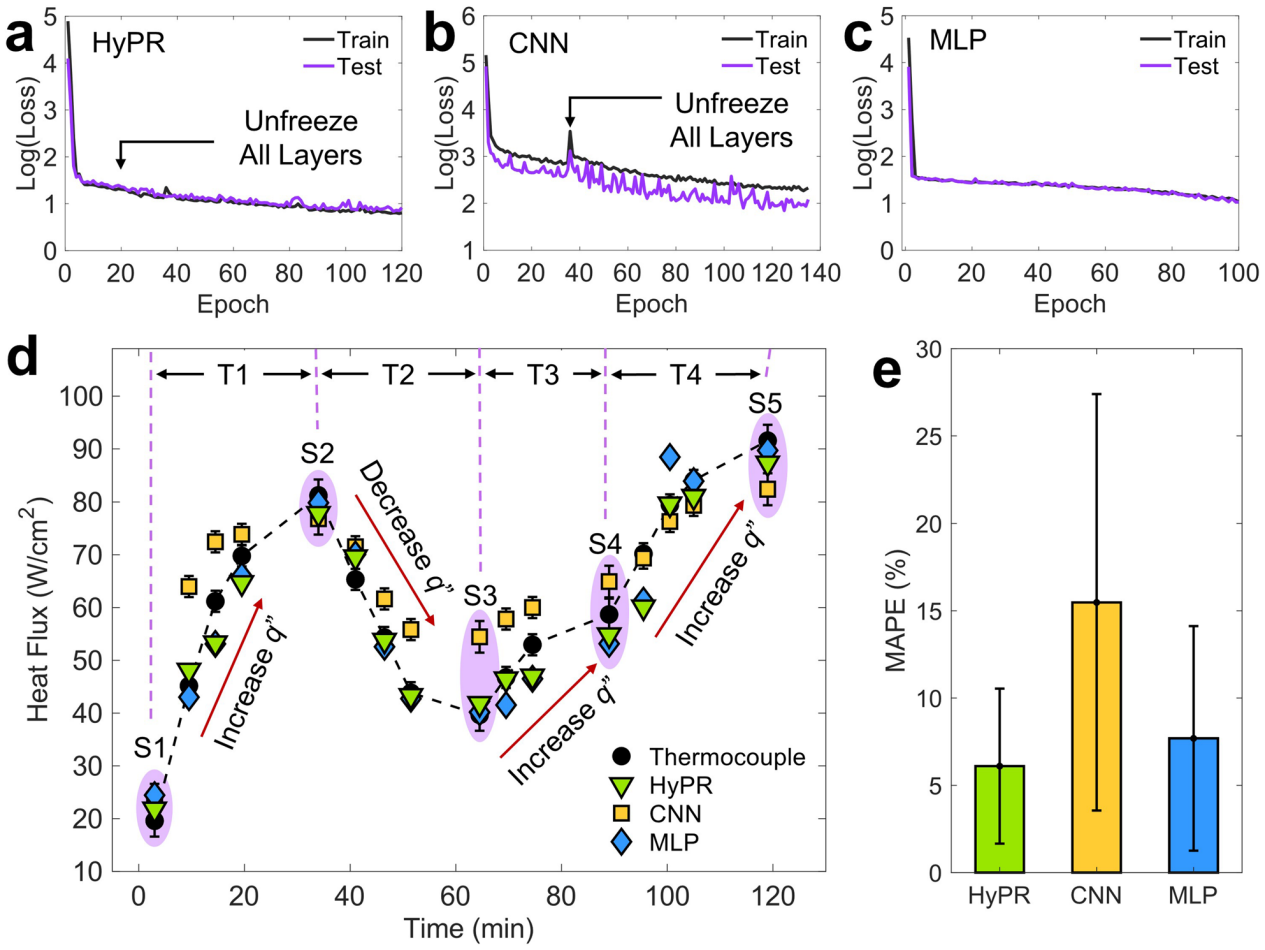
Real-time prediction of boiling heat flux using trained deep learning models. The training results for the (**a**) HyPR, (**b**) CNN, and (**c**) MLP models show all three models can learn well from the teaching dataset with a testing loss of 2.49, 7.11, and 5.36, respectively. The losses are plotted in log scales to show the exponential decay. (**d**) The trained models predict real-time steady state (S1-5) and transient state (T1-4) heat fluxes. The HyPR and MLP models respond well to the increasing and decreasing boiling curves, demonstrating minimal deviations. In contrast, the CNN models follow the general trend of the varying heat flux; however, overpredicts mid-range heat fluxes from 40 to 60 W/cm^y^. Error bars represent the standard deviation of the predictions of all 500 images for each heat flux step. (**e**) Mean absolute percentage errors (MAPE) characterize the realistic prediction accuracy, where the HyPR, CNN, and MLP models report 6%, 15%, and 8% mean error, respectively. The error bars show the standard deviation of MAPE.

The loss graphs in Fig. 5a show that the HyPR model performs well within its trained conditions (experimental sets 1–3). Figure 5b,c compare the training results for isolated image feature-based (i.e., CNN) and bubble-statistic-based (i.e., MLP) prediction models. See Methods Section for detailed training process. For the CNN model, the test loss is relatively lower than the train loss and display more noise compared to the other models. The high train loss with respect to test loss is attributed to data augmentation being applied only on the train dataset, making test dataset images easier to predict. On the other hand, the hybrid model shows much smoother decay than CNN models, verifying the effectiveness of using combined features. The testing loss for the HyPR, CNN, and MLP models are 2.4, 7.1, and 5.3, respectively. While all three models test with relatively low losses, it is imperative to further examine whether the model can realistically generalize to independent experimental sets by using the validation dataset.

### Real-time prediction of boiling heat flux

By using the validation dataset, we compare the real-time boiling heat flux prediction by using all three models with heat flux calculations based on thermocouple measurements. We note that the power input during the validation dataset boiling experiment is spontaneously increased or decreased for five heat flux steps (S1-5). Between steady states, transitional states (T1-4) are also measured to confirm the models’ ability to identify real-time boiling heat flux changes. In Fig. 5d, the prediction values of HyPR, CNN, and MLP models are indicated by using green, yellow, and blue markers, respectively, and the thermocouple measurements are shown in black circles. While all three models predict the general increasing- or decreasing-trend of the new dataset, the CNN model deviates the most from thermocouple measurements and overpredicts mid-range boiling heat fluxes, which are 50–80 W/cm^2^. The error bars in Fig. 5d indicate the standard deviations of heat flux measurements and model predictions. The errors from thermocouple measurements are translated to real-time heat flux changes during transitional steps and measurement uncertainties. The errors from the model predictions represent prediction fluctuations caused by the model making predictions for individual images. The prediction fluctuations of HyPR model are greatly minimized to near nullity, supporting the hypothesis that the bubble statistics features function as prediction guidelines for the hierarchical image feature-based predictions. It should be noted that the prediction fluctuations are relatively high for the CNN models because they operate on an individual image-basis. In contrast, the prediction fluctuations of MLP model cannot be calculated because the MLP model predictions are based on averaged numerical bubble statistics.

The prediction accuracy can be quantified by calculating the mean absolute percentage error (MAPE), which is defined as:

1 $$MAPE= \frac{1}{n}\sum_{i=1}^{n}\left|\frac{{q"}_{measured}-{q"}_{predicted}}{{q"}_{measured}}\right|\times 100$$

where ${q"}_{measured}$ is the thermocouple-based reading and ${q"}_{predicted}$ is the model’s prediction. The absolute value in this calculation is summed for every predicted feature set and is divided by the total number of images *n*. Figure 5e shows the MAPE values for each model, where the HyPR model shows a minimum MAPE of 6%, while the predictions models using CNN and MLP show 15% and 8% MAPE, respectively. The error bars show the standard deviation of MAPE over all heat flux steps. The HyPR model exhibits relatively smaller deviations than the MLP model while both models exhibit similar MAPE. This suggests that the use of hierarchical image features in the HyPR model positively impacts the prediction capability of the hybrid model. Generally, MAPE is known to decrease as the training dataset size increases at the expense of increased training cost. Despite the advantages of using a large image dataset, we intentionally train our model on a small (250 image per class) dataset while achieving similar MAPEs compared to the recent work with 91% dataset size reduction^50^, which confirms that the collective effectiveness of coupling multiple learning techniques (e.g., CNN, Mask R-CNN, MLP, fine-tuning, data augmentation, etc.) performs well. It should be noted that the increases in the dataset size will further improve the MAPE by providing extra learnable image and bubble statistic features with additional computational cost.

## Discussion

The proposed model has a great potential to be customized or upgraded to perform different tasks that account for various experimental environments (i.e., surface characteristics, experimental setup, boiling conditions, and others). For example, our boiling curves in Fig. S3 confirm the formation of different bubble statistics depending on surface characteristics. The nanostructured surface shows larger bubbles as well as smaller bubble count compared to the plain surface at a given heat flux. Such differences in bubble statistics can be incorporated into new models. Furthermore, object tracking modules^58^ will enable the model to collect detailed descriptions about spatiotemporal features (e.g., bubble growth, trajectory, surface interactions, departure frequency, and departure velocity) for different surfaces, which will help retrain surface-dependent prediction models. Similarly, the model has potential to identify the level of surface deterioration. One of the critiques of using micro/nanostructures in boiling is that they are easily damaged after long periods of intensive boiling. Therefore, by correlating the surface characteristics and bubble features, the model may be further trained to identify and even predict bubble statistics changes as the surfaces degrades. Another example includes the auto-correlation associated with experimental setup. For instance, automatic distance and angle estimation modules can potentially liberate the imaging distance and angle by factoring in size and angular compensation variables.

Perhaps more importantly, the use of deep learning framework can be resource effective, in experimental and computational manners. For instance, visualization-based methods are remote, which means that the measurements can be conducted over multiple boiling setups with minimum space requirements. Furthermore, the presented method is cost-effective. Conventional methods using thermocouple and electrical power input setups require wired attachments (i.e., probes and multimeters) while IR cameras need dichroic mirror fixture stages and can only conduct bottom-to-top imaging. In many cases, these attachments substantially increase the costs of boiling devices at both lab and commercial scales. In addition to the space and cost considerations, the learning framework through the image automation significantly saves computational time to analyze large-size datasets by synchronizing image data with the measured values. While high-resolution images are extremely memory-expensive, the transfer learning and data augmentation techniques can reduce the required image dataset size and model training time. The resource-effective framework demonstrated here will help describe other types of image-based transport phenomena to impact the heat transfer community.

## Methods

### Experimental setup

We collect high-fidelity bubble images from four consecutive pool boiling experiments using the setup shown in Fig. 2a. The pool boiling rig mainly consists of the boiling surface, a heating block, a data acquisition device connected to thermocouples, and a high-speed camera. The boiling surface is a 1 cm × 1 cm plain copper sample, which is soldered and left attached on a custom-built copper heating block in all measurements to ensure consistent thermal contact resistance. The boiling surface is cleansed for 5 min before each experiment via a piranha solution. The heating block consists of four cylindrical cartridge heaters, which are heated by AV voltage regulator (Variac Transformer), where an insulating glass wool thoroughly encloses the copper block to promote one-dimensional thermal conduction. The generated heat flux is calculated by taking the average heat flux *q*″ = *k*Δ*T/L* measured from four K-type thermocouples positioned incrementally along the copper heating block where *k* is the thermal conductivity and *ΔT* is the temperature difference measured between a prescribed distance *L.* The uncertainty of thermocouple measurements is ± 1 °C, which leads to an estimated uncertainty of 2.2% at the maximum heat flux by using the law of propagation of uncertainty (see Supplementary Information). The train and test dataset heat fluxes are measured only during steady states while the validation dataset includes both steady and transitional state measurements to demonstrate real-time prediction. Furthermore, the input heat fluxes in the validation dataset are arbitrarily raised and lowered to test the model’s robustness. A data acquisition device (Labjack U6) records temperatures for approximately 3 min during both steady and transitional states. Above the boiling surface, a transparent guard heater-installed boiling chamber maintains degassed DI water in saturation conditions by receiving signals from a PID controller.

### Real-time data acquisition

Pool boiling images and videos are obtained via a high-speed camera (FASTCAM Mini AX50). Since high resolution images convey important bubble statistics in relation to the boiling heat flux, we set the image resolution to 1024 X 1024 pixels in this study. To improve the imaging quality, a light diffuser is placed opposite from the camera to evenly distribute background lighting (Fig. 2a). High speed imaging of 2,000 fps improves the image quality even further by reducing motion blurs. On the other hand, high-speed imaging can produce overly correlated image datasets if captured in high frame sequences. Highly sequential image datasets, in turn, risk being biased towards only a few numbers of bubbles and requires unnecessarily many images to increase the dataset diversity. To minimize potential bias to the identification process caused by highly correlated frame sequences^49,50^, we capture images at random time frames (i.e., randomized imaging) for a duration of 30 s, as shown in Movie S1. The structural similarity index (SSIM) confirms the image dataset correlation by comparing two images, where SSIM = 1 corresponds to identical images and SSIM = 0 indicates completely uncorrelated images. The SSIM plot in Fig. S4a shows that the randomized imaging dataset display relatively lower SSIM indexes than the sequential imaging dataset. The higher correlation of sequential images becomes clearer in the histogram plot (Fig. S4b), where a greater number of high SSIM indexes are observed. On the other hand, randomized images are well distributed (i.e., less correlated) and form a gaussian curve with a relatively low mean SSIM index of 0.6. Therefore, randomized imaging techniques are employed to collect 250 images for each heat flux step, which provides the total of 3,250 images to train the model.

### Datasets

We split the collected images into a train, test, and validation set. Among the four boiling experiments, the images collected from the first three experiments are divided into 80% train and 20% test datasets. Train sets are labeled with heat flux measurements that provide answers required to train the model. In contrast, test sets consist of unlabeled images from the same experimental pool and verify the model’s ability to predict unencountered images. Unlike the test set, the validation set images are collected from the last, separate experiment and evaluates the model’s ability to generalize towards independent experimental conditions.

### Training mask R-CNN

Mask R-CNN generates pixel-wise masks that can be used to extract bubble statistics for each image (Fig. S5). As a brief description, Mask R-CNN builds on the previous semantic segmentation model, Faster R-CNN^59^, and consists of a backbone neural network architecture Residual Learning Network (ResNet) for deep feature learning and feature extraction. Feature Pyramid Networks (FPNs) improve object representation, while Regional Proposal Networks (RPNs) and Region of Interest Align (RoIAlign) functions, which returns candidate bounding boxes. The bounding boxes are applied with bilinear interpolation to predict pixel-accurate masks. Deeper discussion on the mathematical basis of Mask R-CNN is explained elsewhere^57^.

Being a supervised learning model, Mask R-CNN requires labelled data in forms of pixel-wise image annotations in order to learn. We use a commercial annotation software (Supervisely, San Jose, CA, USA) to manually label 50 arbitrarily selected images from the teaching dataset as shown in Fig. S5a,b. The labelling process is greatly minimized by utilizing data augmentation techniques, which increases the generalizability of the model by randomly transforming the original data into new, increased, and slightly modified versions (Fig. S6)^60^. The augmented dataset consists of 704 images where 80% (564 images) are used as a training set and 20% (140 images) are used for testing. In this paper, we initialize the model using weights pre-trained on the Microsoft Common Objects in Context (MSCOCO) dataset^61^. Mask R-CNN trains for a total of 100 epochs using stochastic gradient descent with a learning rate of 1e-3 and momentum of 0.9. A checkpoint at each epoch saves the model’s state for optimal model selection. The training results in Fig. S5c show that training and test loss both decrease, with a minimum test loss of 0.09 at epoch 98, which has been selected for this study. Fig. S5d–g displays the resizing, mask prediction, and overlay process of one example image tested on our trained Mask R-CNN model. A real-time prediction of sequential images is presented in Movie S2. Each image describes individual bubble size and count information that are automatically extracted.

### Training HyPR, CNN, and MLP models

We fine-tune the HyPR model on ImageNet^62^ with an Adam optimizer at a learning rate of 1e-3 for 20 and 100 epochs before and after unfreezing the neural network layers, respectively (Fig. 5a). In order to train isolated CNN and MLP models, we configure their fully connected (FC) layers to have 1 output followed by a linear activation function as shown in Fig. S7. The CNN model is fine-tuned with identical settings as the HyPR model, but with learning rates of 4e−5 for 35 epochs before unfreezing all training layers (Fig. 5b). After stabilization, the model continues to train with all layers unfrozen for 100 epochs with learning rates of 1e−3 (Fig. 5c).

## Supplementary information

Supplementary Information.

Supplementary Movie S1.

Supplementary Movie S2.

## Data Availability

The authors declare that all boiling data and codes supporting this study are available from the corresponding author upon reasonable request. All other data supporting this study are available within the article and its Supplementary Information file.
