# Supplementary material for: Deep learning predicts boiling heat transfer
Source: Sci Rep. 2021 Mar 10;11:5622. doi: 10.1038/s41598-021-85150-4 (PMC7970936; doi:10.1038/s41598-021-85150-4)
Supplement: Supplementary file 1 — Supplementary Information. [file 41598_2021_85150_MOESM1_ESM.pdf]

Supplementary Information for

Deep Learning Predicts Boiling Heat Transfer

*Youngjoon Suh, Ramin Bostanabad, and Yoonjin Won**

Department of Mechanical and Aerospace Engineering
University of California, Irvine
5200 Engineering Hall, CA 92617-2700, USA

*Corresponding Author
Name: Yoonjin Won
Email: won@uci.edu
Telephone: +1 949-824-3414
Location: 4200 Engineering Gateway

S1. Fine-Tuning Deep CNN

Updating and retraining a network on pre-trained weights generally help the training process, even if the weights are trained on images with dissimilar classes, because they usually share many lower-level image features involving edges, textures, and shapes[1]. We utilize model weights pre-trained on ImageNet[2], a vast dataset of over 15 million labeled high-resolution images. As shown in Fig. S1a, the default pre-trained VGG16 architecture consists of 16 weight layers (convolution and FC layers) followed by a softmax function typically used for image classification. Custom FC layers with a single output node and linear regression layer replaces the original FC softmax classifier layer, thereby designating a new task (bubble recognition) to the model and enabling continuous quantity prediction (Fig. S1b). To prevent risk of destroying already learned layers, we initially freeze the early neural network layers and train the model at a low learning rate (Fig. S1c). After the stabilization, we unfreeze the layers and continue training until appropriate accuracies are achieved (Fig. S1d).

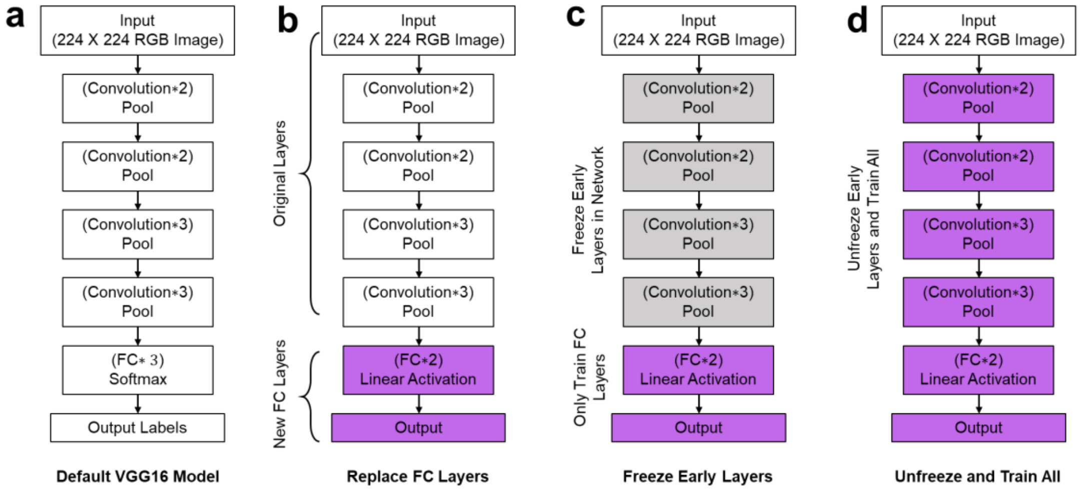

Fig. S1. Fine tuning VGG16. a The original VGG16 network architecture is simplified by boxing convolution layers between every pooling. **b** The fully connected (FC) layers are replaced with customized FC layers followed by linear activation. The new FC layers start with a 512 neurons FC layer followed by a dropout function of 0.5 for regularization. Then, the output is set to a singular node and applied with linear activation for regression problems. **c** The substituted FC layers are totally new and random, risking to destroying already learned features. Therefore, the first few epochs are run while freezing early layers (gray) in the network to prevent backpropagation after the FC layers. **d** After the calculations stabilize, all layers are unfrozen and continue to learn at small learning rates until sufficient accuracy is obtained. The purple color represents layers that are being updated.

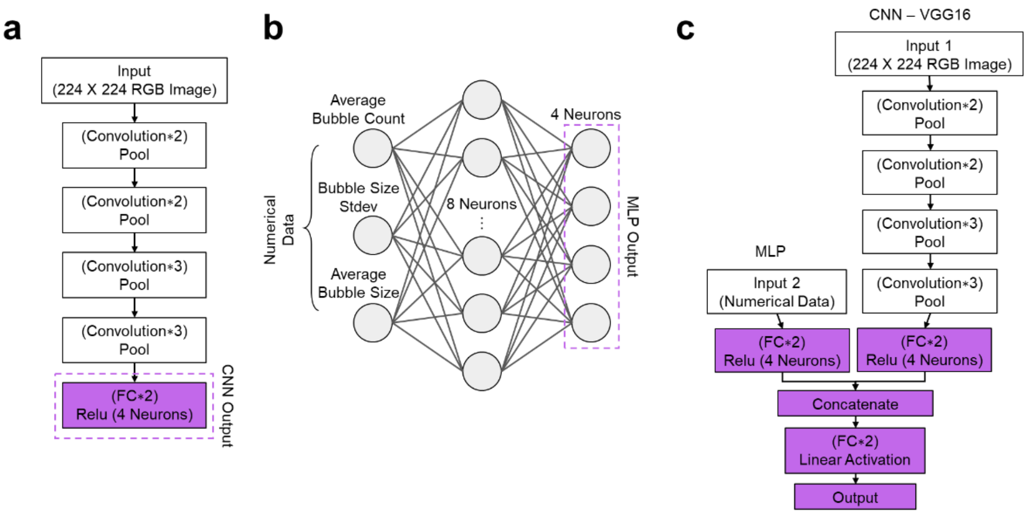

Fig. S2. HyPR architecture. a The convolution neural networks (CNN) model and **b** custom multi-layer perception (MLP) neural networks are configured to have 4 output neurons. The output neurons are compact representations of features reduced from the input and can be modified to help the solution converge. **c** Then, the CNN and MLP outputs are concatenated and applied with a linear activation function to predict the output boiling heat flux. The purple color represents layers that are being modified.

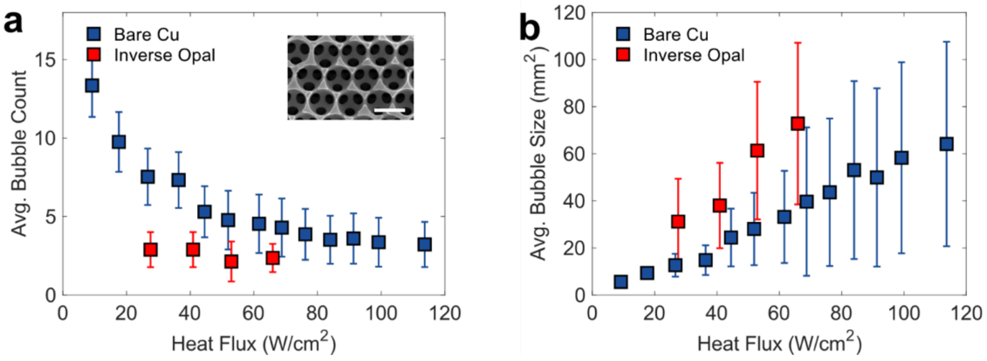

Fig. S3. Bubble characteristics of bare and nanotextured surfaces. a The average bubble count for the nanotextured substrate is significantly lower than the bare substrate even at low heat fluxes, which suggests active bubble coalescence. The inset shows scanning electron microscope images of the nanotextured structure where the scale bar is 1 µm. **b** The average bubble size on the nanotextured surface is relatively larger than bare substrate but increase at similar slopes. Nanotextured surface fabrication procedures are described elsewhere[3].

S2. Uncertainty Analysis for Pool Boiling Experiment

The uncertainties were computed by using the law of propagation of uncertainty. The heat flux $q'' = k\Delta T/L$ is a function of temperature gradients, material properties, and thermocouple positions. Specifically, q'' is calculated by averaging the q'' obtained from thermocouples 1 – 4:

$$q'' = k\left[\frac{\left(\frac{T_1 - T_2}{L_1}\right) + \left(\frac{T_2 - T_3}{L_2}\right) + \left(\frac{T_3 - T_4}{L_3}\right)}{3}\right] \tag{S1}$$

where $T_{i=1,2,3,4}$ are the temperature readings from the four thermocouples used in the experiment, k is the thermal conductivity, and $L_{i=1,2,3}$ are the distance between thermocouples.

We focus on the uncertainties caused by thermocouple readings ($U_T = \pm 1.1^{\circ}C$) by assuming that the thermal conductivity remains constant during experiments and that positional errors are minimized. Therefore, the uncertainty of the heat flux becomes:

$$U_{q''} = \sqrt{\left(\frac{\partial q''}{\partial T_1}U_T\right)^2 + \left(\frac{\partial q''}{\partial T_2}U_T\right)^2 + \left(\frac{\partial q''}{\partial T_3}U_T\right)^2 + \left(\frac{\partial q''}{\partial T_4}U_T\right)^2} \tag{S2}$$

By solving for Eqn. (S2), an uncertainty of approximately 2.2% is calculated for the maximum heat flux.

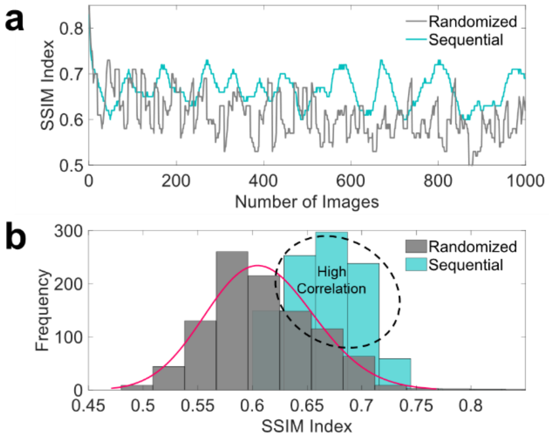

Fig. S4. Randomized and sequential imaging comparison. The structural similarity index (SSIM) compares the similarity between two images, where 0 corresponds to no similarity and 1 infers identicalness. **a** Randomized imaging diversifies the image dataset. SSIM plots the difference between 1000 images and one reference image (image number 0). **b** Randomized images have lower and more widely distributed SSIM and therefore less bias.

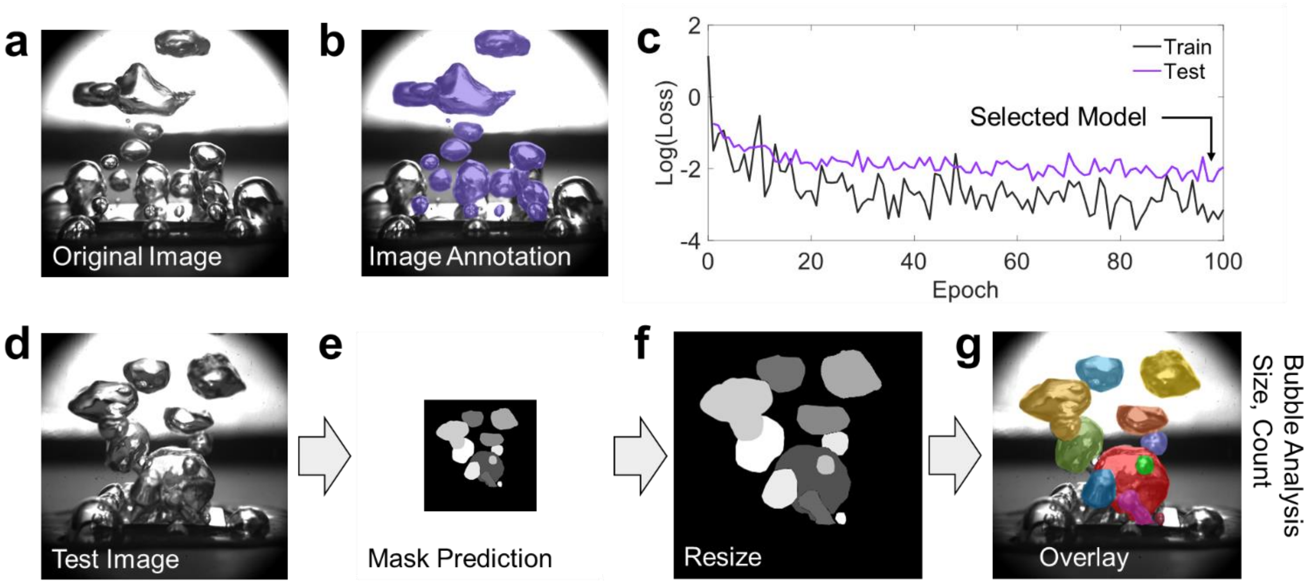

Fig. S5. Instance segmentation via Mask R-CNN. To train Mask R-CNN on a new task, images must be manually labeled (i.e., annotated). The representative example images show **a** before and **b** after annotation. **c** The training results for Mask R-CNN show an optimal checkpoint at epoch 98 which is selected to analyze bubbles throughout this study. **d** The images are processed through a series of steps, including **e** masking, **f** resizing, and **g** final overlaying to produce the segmented image output. The output enables to identify bubble statistics (i.e., bubble counts and size per frame) used as input features.

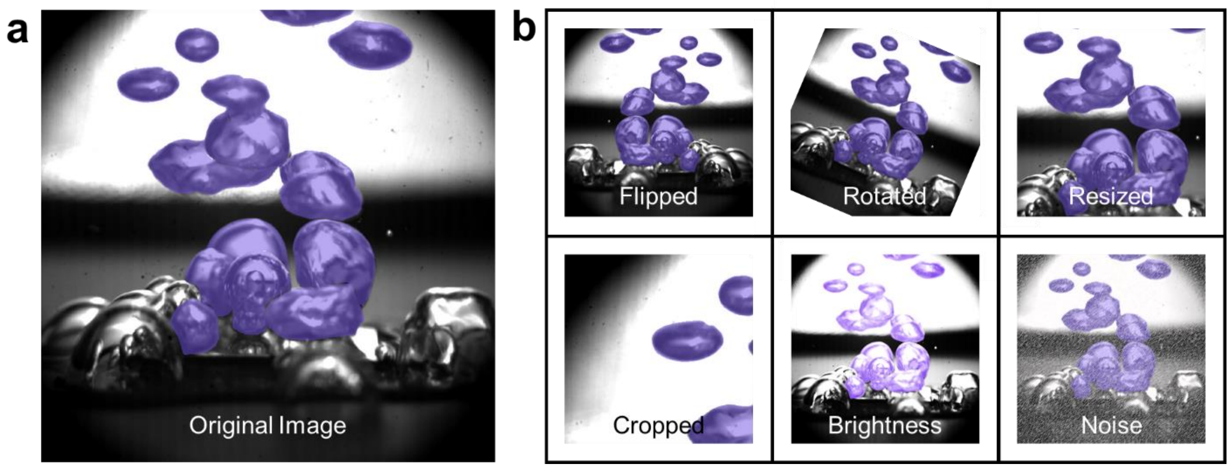

Fig. S6. Data augmentation techniques. a An example image of an annotated image used for Mask R-CNN training is shown. **b** Data augmentation diversifies the training dataset by flipping, rotating, resizing, cropping, changing the brightness, and introducing noise to the original image. It should be noted that significantly less aggressive data augmentation is employed when training CNN and CNN+MLP models to prevent invalid inputs.

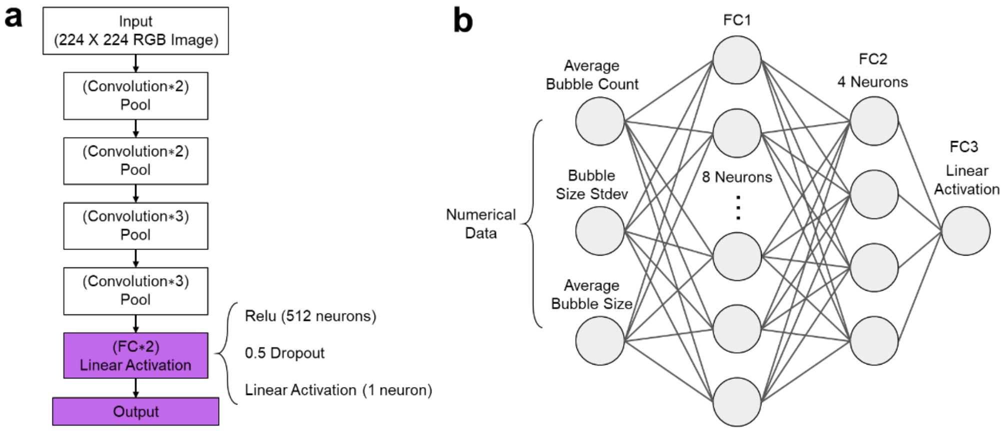

Fig. S7. Configurated CNN and MLP architecture. a The final FC layer of the isolated CNN model is set to 1 neuron followed by linear activation to produce the heat flux output. The input to the CNN model is RGB boiling images. On the other hand, **b** The input to the MLP model includes the average bubble count, bubble size standard deviation, and average bubble size. The FC layers are set to have 8, 4, and 1 hidden unit (i.e., neurons), respectively followed by linear activation.

References

1 Norouzzadeh, M. S. *et al.* Automatically identifying, counting, and describing wild animals in camera-trap images with deep learning. *P Natl Acad Sci USA* **115**, E5716-E5725, doi:10.1073/pnas.1719367115 (2018).

2 Deng, J. *et al.* ImageNet: A large-scale hierarchical image database in *2009 IEEE conference on computer vision and pattern recognition.* 248-255 (2009).

3 Pham, Q. N., Suh, Y., Shao, B. & Won, Y. Boiling Heat Transfer Using Spatially-Variant and Uniform Microporous Coatings. *Proc Asme Int Tech C* (2020).
